# Supplementary material for: Differential effects of BCG-Russia and BCG-TICE on trained immunity: Potential implications for bladder cancer immunotherapy
Source: PLoS One. 2026 Sep 11;21(9):e0357950. doi: 10.1371/journal.pone.0357950 (PMC13567787; doi:10.1371/journal.pone.0357950)
Supplement: Supplementary file 2 [file pone.0357950.s004.docx]

**Review PONE-D-26-23397**

1. A total of 36 mice were used. How many were in the control group, and how many were assigned to the BCG TICE and BCG Russia strains? This information is not mentioned in the Methods section and should be included. How many females and how many males were used? Please also specify the number of animals in each group. The study design is not clear; it is only generally stated that there were 36 mice aged between six and eight weeks
2. In **Figure 3**, the term "strain" should be changed to the plural form, "strains," Fig 3. Exemple: Effects of BCG strains on cytokine production by bone marrow-derived macrophages (BMDMs) **or** Effects of BCG strains (TICE and Russia) on cytokine production by bone marrow-derived macrophages (BMDMs)
3. In **Figure 3** you comment: “Data points represent values from independent biological replicates (all male)” Why are they all male? What about the females? Were all 36 mice evaluated here?
4. In **Table S1.** Raw values of plasma cytokine concentrations HAY PBS (n=5), BCG-Russia (n=6) y BCG-TICE (n=4). The authors should report the total number of mice used in the study and explain why only 4 PBS mice were included in the IL-2 evaluation.

The sample sizes also vary for TNF-α: there are 4 mice in the PBS group, 5 in the BCG Russia group, and 5 in the BCG-TICE group.

Overall, a total of 21 mice were evaluated in the BCG-TICE group, 29 mice in the BCG Russia group, and 23 mice in the PBS group. The authors should clarify these discrepancies and provide a clear accounting of all animals included in each analysis
