# Supplementary material for: Differential effects of BCG-Russia and BCG-TICE on trained immunity: Potential implications for bladder cancer immunotherapy
Source: PLoS One. 2026 Sep 11;21(9):e0357950. doi: 10.1371/journal.pone.0357950 (PMC13567787; doi:10.1371/journal.pone.0357950)
Supplement: Supplementary file 3 [file pone.0357950.s006.docx]

**Journal Requirements:**

*1. Please ensure that your manuscript meets PLOS ONE's style requirements, including those for file naming.*

We have ensured that the manuscript meets PLOS ONE’s style requirements.

*2. Please note that PLOS One has specific guidelines on code sharing for submissions in which author-generated code underpins the findings in the manuscript. In these cases, we expect all author-generated code to be made available without restrictions upon publication of the work. Please review our guidelines at https://journals.plos.org/plosone/s/materials-and-software-sharing#loc-sharing-code and ensure that your code is shared in a way that follows best practice and facilitates reproducibility and reuse*.

No code was generated for this submission.

*3. In the online submission form you indicate that your data is not available for proprietary reasons and have provided a contact point for accessing this data. Please note that your current contact point is a co-author on this manuscript. According to our Data Policy, the contact point must not be an author on the manuscript and must be an institutional contact, ideally not an individual. Please revise your data statement to a non-author institutional point of contact, such as a data access or ethics committee, and send this to us via return email. Please also include contact information for the third party organization, and please include the full citation of where the data can be found.*

All relevant data generated for this manuscript are included within the manuscript.

*4. If the reviewer comments include a recommendation to cite specific previously published works, please review and evaluate these publications to determine whether they are relevant and should be cited. There is no requirement to cite these works unless the editor has indicated otherwise.*

We added a citation suggested by Reviewer 1, which we agree it is relevant to the manuscript.

**Response to Reviewers’ comments**

**Reviewer #1**

1. *A total of 36 mice were used. How many were in the control group, and how many were assigned to the BCG TICE and BCG Russia strains? This information is not mentioned in the Methods section and should be included. How many females and how many males were used? Please also specify the number of animals in each group. The study design is not clear; it is only generally stated that there were 36 mice aged between six and eight weeks.*

**Response:** We thank the reviewer for this constructive comment. We have updated this sub-section (‘*Mouse Models*’) of the *Methods* with the additional details requested (Lines: 109-115). Additionally, we have revised each figure caption to specify the relevant sample sizes, sex distribution, and treatment group allocation. We believe this information is most clearly communicated by including it in figure captions.

2. *In* ***Figure 3****, the term "strain" should be changed to the plural form, "strains," Fig 3. Example: Effects of BCG strains on cytokine production by bone marrow-derived macrophages (BMDMs)* ***or*** *Effects of BCG strains (TICE and Russia) on cytokine production by bone marrow-derived macrophages (BMDMs)*

**Response:** Revision made as requested.

3. *In* ***Figure 3*** *you comment: “Data points represent values from independent biological replicates (all male)” Why are they all male? What about the females? Were all 36 mice evaluated here?*

**Response:** We agree that this statement would benefit from further clarification. The statement in the *Methods* section that “A total of 36 mice were used” was in references to all studies completed for this manuscript. The revised wording in this section should enhance clarity. As an important ethical consideration, we attempt to minimize animal use wherever possible. However, certain experiments required separate cohorts of mice for data integrity purposes. For example, mice subject to *in vivo* re-challenge (*see Fig. 2*) were not used for BMDM generation due to the potential confounding effects of *in vivo* LPS exposure on *ex vivo* BMDM assays. Consequently, no one figure in the present manuscript includes data from all 36 mice. Nine (out of 36) mice were used to generate the Figure 3 data. Only one sex was included because we had 9 available males at the time of study initiation. However, as you can see from revisions made to the *Methods* section (Line: 113), our studies had an equal sex distribution in aggregate. This demonstrates that are observations were not limited to male mice as similar BCG strain-dependent trends were observed in experiments with primarily female mice (*e.g.,* Fig. 2).

4. *In* ***Table S1.*** *Raw values of plasma cytokine concentrations PBS (n=5), BCG-Russia (n=6) BCG-TICE (n=4). The authors should report the total number of mice used in the study and explain why only 4 PBS mice were included in the IL-2 evaluation.*

*The sample sizes also vary for TNF-α: there are 4 mice in the PBS group, 5 in the BCG Russia group, and 5 in the BCG-TICE group.*

**Response:** We thank the reviewer for identifying this discrepancy and allowing us to clarify. As mentioned in our response to comment #3, the experiment associated with Figure 2 (and Table S1) involved a separate cohort of animals. A total of 15 mice were enrolled, with treatment group allocation as follows: PBS (n=5), BCG-Russia (n=6), and BCG-TICE (n=4). This information is now more clearly specified in the figure caption (Line: 262) and can also be deduced from the data points displayed in the figure. Only four IL-2 values were included for the PBS group because one mouse’s plasma sample contained IL-2 levels below the detectable threshold. Similarly, one mouse’s plasma sample (PBS group) contained TNF-α levels below the detectable threshold. To improve transparency, we have added these values back into Table S1, with a brief explanation in the table caption.

The BCG-Russia sample size for TNF-α is n=5 (not n=6) because one value from this group was excluded following an outlier test, as described in the Figure 2 caption. We have updated the caption so that the relevant group is clearly indicated (Line: 264).

*Overall, a total of 21 mice were evaluated in the BCG-TICE group, 29 mice in the BCG Russia group, and 23 mice in the PBS group. The authors should clarify these discrepancies and provide a clear accounting of all animals included in each analysis.*

**Response:** We thank Reviewer #1 for their valuable feedback. In addressing these comments, we have improved transparency surrounding the distribution of animals across studies by clearly indicating this information in each figure caption, as well as an overall summary in the *Methods* section (Lines: 109-115). Our manuscript includes data from a total of 36 mice, across multiple independent experiments. Overall, a total of 11 mice were evaluated in the BCG-TICE group, 13 mice in the BCG Russia group, and 12 mice in the PBS group.

**Reviewer #2**

1. *The manuscript would benefit from citing clinical evidence involving BCG-Russia, particularly studies evaluating its use in patients, to better contextualize the translational relevance of the observed differences. Recent clinical studies show that BCG-Russia performs comparably to other BCG strains in terms of efficacy as adjuvant treatment for papillary non–muscle-invasive bladder cancer (NMIBC) and for treatment of carcinoma in situ (CIS) of the urinary bladder. These findings should be cited and discussed (http://dx.doi.org/10.5489/cuaj.8552), as the differences observed in the current study do not appear to translate into clinically meaningful differences in efficacy in patient-based studies.*

*Regarding the interpretation that BCG-TICE induces a “broader pro-inflammatory profile than BCG-Russia,” this statement may require refinement. While some inflammatory pathways are indeed more strongly induced by BCG-TICE, the data suggest a more nuanced pattern. Notably, IL-12 levels appear comparable between groups, indicating that not all Th1-associated signals are enhanced in a strain-dependent manner. This is further supported by the observation that several pathways show opposing regulation between strains.*

**Response:** We appreciate this important comment and agree that it is possible that BCG-Russia is therapeutically as effective as BCG-TICE. The study by Flury-Sutter et al., cited by the reviewer, indeed shows that BCG Russia is efficacious. However, that study was a single-arm study not designed to compare the therapeutic effect of BCG-Russia against BCG-TICE directly. The ongoing EVER non-inferiority trial (NCT05037279) will hopefully answer this question. It is also important to point out that our study focuses on describing BCG strain-dependent differences in trained immunity acquired by bone marrow-derived macrophages. It is possible that other cell types, which could possibly mediate the therapeutic effect of BCG, such as neutrophils, do not show strain-dependent differences in trained immunity acquisition. We have added these comments to the manuscript (Lines: 61-66). Regarding the Reviewer's criticism of our statement about BCG-TICE inducing a "broader pro-inflammatory profile than BCG-Russia" based on differential secretion of Th1 cytokines, we agree that our statement describing those findings may be an over-interpretation of the results. Therefore, we have removed the interpretation sentence at the end of that section in Results (Line: 256). However, our conclusion that BCG-TICE induces a stronger inflammatory response is further supported by our Nanostring data showing that, compared with BCG-Russia, BCG-TICE significantly induced the activation of various pro-inflammatory pathways.

2. *What is the rationale for using intravenous stimulation in the experimental design? Given that BCG is most commonly administered intravesically in clinical settings, it would be important to discuss how systemic (intravenous) exposure may influence the trained immunity phenotype observed in BMDMs. Specifically, it remains unclear whether the strain-specific differences reported here would be preserved under intravesical stimulation, where local mucosal immunity, cellular recruitment, and tissue-specific microenvironmental cues play a dominant role. Addressing this point would significantly strengthen the translational interpretation of the findings.*

**Response:** Our method of trained immunity induction is based on a widely used model of BCG-induced central trained immunity (ex. PMID: 29328912, PMID: 0446799, PMID: 40555239). *Central* trained immunity refers to reprogramming acquired at the level of bone marrow progenitor cells, in contrast to *peripheral* trained immunity acquired my circulating monocytes or tissue macrophages. This approach has certain advantages, including strong and consistent induction of trained immunity across experimental animals. There is also limited knowledge surrounding the optimal conditions to experimentally induce trained immunity through the intravesical route. It is understood that NMIBC patients likely experience variable trained immunity acquisition over the course of BCG therapy. Intravenous stimulation also results in central trained immunity being the dominant biological effect of BCG, whereas intravesical administration involves local mucosal immunity, as the reviewer pointed out. Therefore, this approach was preferred because of our study’s specific focus on BCG strain-dependent differences in trained immunity. At the reviewer’s request, we have expanded on the potential influence of administration route on trained immunity phenotypes in the relevant section of the discussion (Lines: 484-503).

3. *It would be important to discuss the molecular and genomic differences between BCG-TICE and BCG-Russia strains, as these are likely to underlie the observed functional divergence. While both belong to the BCG family, they have accumulated distinct deletions and mutations during independent propagation lineages, which may impact antigenic repertoire, cell wall composition, and PRR engagement. In this context, could the phenotype associated with BCG-TICE be partially reproduced by modulating specific innate immune pathways activated by BCG-Russia? For example, it would be valuable to consider whether combinatorial stimulation or targeted agonism of key PRRs (e.g., TLR2/NOD2 axes) could recapitulate aspects of the TICE-associated trained immunity profile, or whether the observed differences are dependent on strain-specific structural and persistence-related properties that cannot be easily mimicked.*

**Response:** We agree the manuscript would benefit from further discussion of this point. We did not provide significant details of the molecular and genomic differences between BCG strains in the first draft because it is highly speculative and additional studies would be needed to determine to specific link between these molecular differences and trained immunity phenotypes. We have included some examples of genomic differences between known immunogenic proteins in the discussion (Lines: 470-478).
